# Personal and Social Responsibility Model: Differences According to Educational Stage in Motivation, Basic Psychological Needs, Satisfaction, and Responsibility

**DOI:** 10.3390/children10050864

**Published:** 2023-05-12

**Authors:** David Manzano-Sánchez, Manuel Gómez-López

**Affiliations:** 1 Department of Didactics of Musical, Plastic and Corporal Expression, Faculty of Education and Psychology, University of Extremadura, 06006 Badajoz, Spain; 2 Department of Physical Activity and Sport, Faculty of Sports Science, University of Murcia, 30720 Murcia, Spain; mgomezlop@um.es

**Keywords:** physical education, innovation, responsibility, elementary education, secondary education

## Abstract

The purpose of this study was to apply the Personal and Social Responsibility Model (TPSR) and verify its effects on responsibility and motivation according to educational stage. For this, teachers from Physical Education and other subjects were trained and a pre-test and a post-test were carried out. The intervention was carried out over five months. The total sample comprised 408 students after the inclusion criteria were applied to the initial sample of 430, including being 192 students from 5th and 6th grade of Elementary (M 10.16; SD = 0.77) and 222 from Secondary (M = 12.86; SD = 0.70), with a confidence level of 95% and 5% error margin. The total number of students in the experimental group was 216, with 192 in the control group. The results reflected improvements in the experimental group in terms of experience motivation, identified regulation, amotivation, autonomy, competence, social responsibility, SDI, and BPNs (*p* < 0.05 and d Cohen > 0.2). The control group did not present differences in any variable. Considering the differences according to stage, the Elementary school group obtained values of *p* < 0.05 and d > 0.02 in experience motivation, amotivation, autonomy, competence, personal and social responsibility, SDI, and BPNs, which was not found in the Secondary school group. It is concluded that the TPSR may be applicable in both Elementary and Secondary schools to improve the motivation and responsibility of students, with the most favorable results for Elementary education students.

## 1. Introduction

The current educational system in Spain seeks to promote responsibility in the classroom, both in Elementary Education (“Develop individual and teamwork habits, effort and responsibility in the study” [1]) and Secondary Education (“Develop entrepreneurial spirit and self-confidence… to make decisions and assume responsibilities” [2]). This same can be seen reflected in the Organic Law 3/2020 of Education [3], which indicates the need to “integrate competences… reinforcing autonomy, reflection and responsibility” in its pedagogical principles.

Responsibility is a variable that has been widely studied for the promotion of healthy habits and behaviors in relation to the intention to be physically active or having fun [4], as well as for the improvement of psychological variables such as self-concept [5,6], school social climate [7,8,9], or resilience [10]. In the same way, the reduction of violence or disruptive behaviors has also been the object of study through the promotion of responsibility [7,11], studies of these variables being essential due to the increase in school violence [12,13].

In turn, responsibility has been widely studied as it relates to intrinsic motivation [7,8,14,15,16,17] and with the basic psychological need for autonomy [18], as well as with the perception of competence and social relationships [18]. These variables are of special interest if we consider that human behavior is largely influenced by motivational factors [19,20]. This is where it is necessary to talk about the existence of theories that have tried to explain the functioning of motivation.

Among these theories, the Self-Determination Theory (SDT) of Deci and Ryan [19,21,22] has been widely studied. SDT is a broad framework for understanding factors that undermine intrinsic motivation, extrinsic motivation, and psychological wellness which are essential to adequate environments [23]. SDT focuses on different types of motivation along a continuum. The first of these is intrinsic motivation, which is a prototypical expression of the active integrative tendencies in human nature. This kind of motivation pertains to different tasks for enjoyment or inherent interest. It is the most suitable for creating new behaviors or improving adherence. On the other hand, extrinsic motivation has three subtypes ranging from more self-determined and autonomous motivation with an internal control (identified regulation) to a more external control (introjected regulation and external regulation), and amotivation (impersonal control). Embedded within SDT is Basic Psychological Needs (BPN) theory, which asserts that humans have different basic psychological needs: autonomy, the need to self-regulate one’s experiences and actions; competence, defined as the basic need to feel effectiveness and mastery; and relatedness, which refers to “feeling socially connected” as an integral member of a social group [19,24].

Following the SDT, Vallerand’s hierarchical model [25] shows that there is a series of social reasons or antecedents, among which is responsibility [14,26,27], that can have influence as precursors in the satisfaction of BPN to promote a more self-determined type of motivation (intrinsic motivation and identified regulation) and physical activity among students [28].

One of these precursors may be responsibility, which is considered essential for the development of children and young people [29]; according to Hellison [30], in order to function adequately in society, people must learn to be responsible with themselves and with others. On the other hand, the values it transmits, such as promotion of social relationships or autonomy, are essential for a proper future of children and adolescents [31].

Another model to consider is the Personal and Social Responsibility Model (TPSR) [30], considered as one of the most effective approaches in terms of developing values [32]. This model has shown to be adequate for the improvement of numerous variables, and a few very recent investigations have analyzed the potential of the TPSR model following the SDT. Not only has it seen adequate results in promoting social responsibility [32,33,34,35,36,37,38,39] or personal responsibility [32,33,34,35,36,37,38,39], but this model is also suitable for increasing more self-determined motivation [8,18,36,40] and BPN, especially autonomy [35,39], and also the satisfaction of autonomy, competence, and relationships [7,18,41].

Studies prior to 2019 applied TPSR in different contexts, but it has never been applied in the educational context outside of Physical Education (PE) or extracurricular activities, a suggestion that Llopis-Goig made in his study in 2011 [42]. However, the results obtained by research in the general educational context together with PE [43,44,45,46,47] are able to be extended into other studies such as the current one. In turn, although there are many studies on the TPSR a dating back to the 80s, there are few investigations that have been carried out considering the SDT or, above all, differentiating according to educational stages. The TPSR model has been field-tested for 40 years in different grades from Elementary and Secondary education [43] but very little research has been done differentiating both stages. Only the study by Sánchez-Alcaraz [33] analyzes the differences in the application depending on the educational stage. For this reason, it is considered of great interest to verify the effects of the TPSR model applied in the Elementary and Secondary school stage, taking into account whether the effects are positive in both cases.

For all these reasons, the main objective of this study was to verify the effects of the TPSR model on motivation, responsibility and autonomy, perception of competence, and relationship needs in students. In turn, the second objective is to verify whether the application of the TPSR in the experimental group produced different results according to educational stage.

Based on the review carried out, it is hypothesized that: (1) The application of the TPSR in the general educational context and in PE will allow an improvement in internal motivational values, satisfaction of basic psychological needs, and the responsibility of the students; (2) Elementary school students will obtain higher values in the pre-test in the variables studied; (3) Elementary school students will have higher improvements than Secondary school students when applying the TPSR.

## 2. Materials and Methods

### 2.1. Design

This study is a quasi-experimental pre–post study [48] with a quantitative design [49]. In order to analyze the variables, a multiple-choice questionnaire were applied to the students before and after intervention. The study was conducted according to the guidelines of the Declaration of Helsinki [50] and approved by the Ethics Committee of the University of Murcia (1685/2017).

### 2.2. Participants

The sample consisted of 430 students from the fifth year of Elementary school to the fourth year of Secondary school from three public centers located in Murcia (Spain). After omitting the questionnaires that were not completed and implementing the statistical procedures to detect atypical cases (Mahalanobis’s distance), the final sample was 408 students. There were 192 students from Elementary school (M = 10.16; SD = 0.77) and 222 from Secondary school (M = 12.86; SD = 0.70). The experimental group was made up of 216 students, and the control group 192 students.

All students completed an informed consent form that they passed on to their parents to participate in the study. Surveys indicated that these were completely anonymous in order to develop the methodology. In turn, they signed an agreement that the filming carried out could not be publicly displayed and would only be carried out for research purposes.

### 2.3. Measures

A closed question test with two parts was used in the present study: a first section with socio-demographic questions (gender and date of birth) and a second part with different aspects that were analyzed in this study. Motivation towards Education Scale: a continuous measure of motivation. The values ranged from a more intrinsic motivation to the most external causes, and finally amotivation. The Échelle de Motivation in Education was used [51] and validated by Nuñez et al. [52]. The questionnaire consisted of seven subscales: intrinsic motivation to know, to accomplish and to experience sensations (the most internal regulation); identified regulation (to obtain something external with which I identify); introjected regulation (e.g., “to prove myself that I am an intelligent person”); external regulation (e.g., “to get a more prestigious job”), and amotivation (a complete lack of motivation). The questionnaire was composed of 28 items preceded by the sentence “I go to school/high school because…”, with a seven-point Likert-type scale, from 1 (totally disagree) to 7 (totally agree), and was distributed into seven subscales, five of them containing four items and two of them containing three items. The internal consistency was α = 0.810; 0.752; 0.822 for intrinsic motivation to know, to experience, and to accomplish, respectively; identified regulation (α = 0.704); introjected regulation (α = 0.724); external regulation (α = 0.684); and amotivation (α = 0.777). The only variable that had less than 0.70 (0.684) was external regulation, an acceptable value according to Curran et al. [53]. Furthermore, the Self-determination index (SDI) was applied using the formula ((intrinsic motivation × 2 + identified regulation) − (introjected regulation + external regulation)/2 − (amotivation × 2).

Personal and social responsibility: The Spanish translation developed by Escartí et al. [16] of the personal and social responsibility questionnaire by Li et al. [54] was employed. The questionnaire is made up of two factors of seven items each: personal responsibility (e.g., “I want to improve”), and social responsibility (e.g., “Respect for others”). It was answered through a Likert-type scale with six response options, ranging from (1) totally agree to (6) totally disagree. The internal consistency coefficients for social responsibility were α = 0.801 for social responsibility and 0.782 in the case of personal responsibility.

Basic psychological needs. The purpose of this questionnaire was to measure the satisfaction of the three basic psychological needs. The questionnaire by Moreno et al. [55,56] was used. It is composed of 18 items, six to assess each of the needs: competence (e.g., “I am confident to do the most challenging exercises”), autonomy (e.g., “I think I can make decisions in my workouts”), and relationship with others (e.g., “I feel close to my training partners because they accept me as I am”). The previous sentence is “In my trainings…” and the answers are collected on a Likert-type scale, whose score ranges from 1 (totally disagree) to 5 (totally agree). The alpha-cronbach’s values were 0.717 (autonomy), 0.666 (competence), and 0.757 (relationship). We also used the Basic psychological needs index (BPNs) composed of the means of three variables (autonomy + competence + relationship)/3.

### 2.4. Procedure

The study began after making the appropriate contacts with those responsible for the different centers and accepting participation in the study. The interventions were carried out for a total of five months (second and third academic trimester), in which the participating teachers were previously trained in two 4 h sessions in the TPSR methodology. Questionnaires were completed before carrying out the intervention with the students in PE classes. Before beginning the research, the headmaster informed the parents of the study after holding an informational meeting with the volunteer teachers who participated in the study. The requirement was to spend 60% of the total class time applying the TPSR methodology, in which the PE teacher always participated, along with other school subjects. The questionnaires were completed in the classroom itself, in a quiet environment, taking a total of approximately 20 to 30 min to complete (depending on the age of the participants and their level of understanding).

#### 2.4.1. Personal and Social Responsibility Model

**Figure 1:**
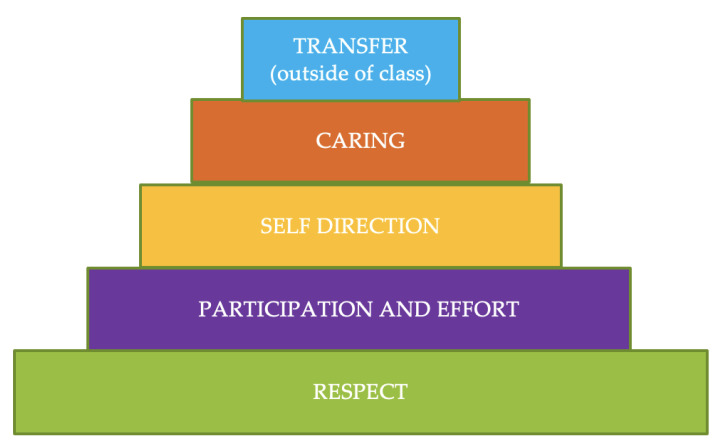
Responsibility levels of TPSR.

The study consisted of the implementation of Hellison’s [30] TPSR in the experimental group in an educational context. This model focuses on providing adolescents with experiences that allow the development of personal and social responsibility skills, progressively giving up responsibility, and has three basic pillars. The first is responsibility levels (Figure 1), which progressively increase to achieve educational values such as respect, effort, autonomy, and help to others. Second is the session structure. The five original parts of Hellison [57] were reduced to four, following other studies applied in this context [58]. The other parts of the session were (a) Relational time: The teacher interacted with their students to create an adequate climate and explain the purpose of the session and tried to put responsibility into practice, introducing the level to be worked at during the session; (b) Action responsibility: the responsibility level selected for the session was embedded in all the tasks; (c and d) Group meeting and co-evaluation: when a session ended, the students and teacher shared their perceptions about responsibility, and students evaluated their classmates’, teacher’s, and their own behavior (with the “thumbs up” strategy) [35]. Finally, the third pillar is the resolution of conflicts. During the classes, strategies were found to resolve conflicts of an individual type (e.g., strategy of five clean days) and group (e.g., the law of the grandmother) [59].

#### 2.4.2. Implementation Fidelity

The need for a sustained implementation of the TPSR as well as the establishment of clear guidelines for its proper development make the concept of “continuous professional development” (CPD) [60] an essential aspect to guarantee success in teacher training and in obtaining results. Some of the most valuable tools for the CPD include systematic observation, and in the case of the TPSR, the so-called “Tool to Evaluate Education Based on Responsibility” (TARE) was used, which is very beneficial to adequately develop any investigation using the TPSR [61,62]. To ensure that the model was being properly applied, different sessions were filmed, each with different students. These were analyzed using the first TARE version (strategies used by teachers). At five-minute intervals, the observer noted the absence or presence of the categories into which the measures were divided (e.g., “Example of respect”). An observer was trained in the use of TARE and how to analyze the different sessions, providing feedback to the teachers with the positive aspects and how to improve for the next session. The teachers were invited to participate in different continuous monthly training sessions to clarify doubts and reach a consensus on aspects that may have raised doubts.

### 2.5. Statistical Analysis

For data analysis, the statistical program SPSS v.24.0 (Statistical Package for the Social Sciences, SPSS Inc., Chicago, IL, USA) was used, and a descriptive and inferential analysis of the results of the initial and final questionnaires of the participants was carried out to know the effect of the intervention on motivation, basic psychological needs and responsibility. Previously, the database was cleaned to detect atypical cases and two participants were eliminated due to having a *p* value < 0.01 in the Mahalanobis test. In addition, the values of skewness and kurtosis were analyzed, less than two in asymmetry and seven in kurtosis [53] being considered appropriate. Next, Cronbach’s Alpha test was used to analyze reliability, obtaining a value of (>0.70) in the different variables.

We checked the results with parametric and non-parametric tests due to the lack of normality of the data, obtaining similar results. To the parametric test a repeated measures analysis including both groups and the variables at two time points was made. For the non-parametric tests, we divided the data into groups and used the U-Mann–Whitney Test (differences between groups) and Wilcoxon Test (differences in two time points) to compare the pre-test and post-test variables. All the statistics followed significance levels of *p* < 0.01 (**) or *p* < 0.05 (*). Additionally, the intervention effect size was estimated using Cohen’s d [63]. The effect size was considered small when it was 0.2–0.49, medium when it was 0.50–0.8, and large when it was greater than 0.8, following Cohen.

## 3. Results

### 3.1. Descriptive Values and Correlations

Table 1 shows the descriptive analyses of the different variables under study. The correlation between the variables was positive in all cases (*p* < 0.01), except for amotivation, which was negative. In turn, the asymmetry and kurtosis values were checked, showing adequate values in all cases, except for amotivation (2.408), with values of 2 being considered adequate according to Curran et al. [53].

### 3.2. Results of the Intervention

In order to know the effect of the TPSR model on the students (Table 2), the Wilcoxon non-parametric test was carried out for both the control group and experimental group. First, we checked the base point to both groups on pre-test. In this sense, it is noteworthy that the values were *p* < 0.05 for all variables using the Mann–Whitney U test in favor of the experimental group, except for amotivation, which was lower, and for introjected regulation, where there were no statistically significant differences. Similar results were obtained in the post-test, with the highest values in the experimental group.

We used the Wilcoxon test to see the difference between groups after the intervention (control group or experimental group). After that, the results were verified using parametric procedures (repeated measures tests and MANOVA), and the results were similar.

We considered how different variables changed between two measures (pre-test and pos-test). In this sense, the control group did not show statistically significant differences in any of the variables between the pre-test and the post-test; only personal responsibility was close to being significant (*p* = 0.063), and all values of the d-Cohen effect were <0.2. On the other hand, the experimental group showed statistically significant differences in knowledge motivation, experience motivation, identified regulation, introjected regulation, basic psychological needs for autonomy and competence, social responsibility, BPNs, and SDI, as well as in amotivation, which was significantly lower (*p* < 0.01), along with Accomplish motivation (*p* < 0.05). In this sense, the effect looks small in most variables, except for Knowledge and Accomplish motivation, and introjected and external regulation, where the value was <0.2. There were no differences in Personal responsibility, External regulation, or Relationship, but personal responsibility had a small effect size (>0.2). Finally, when comparing the effect size of intergroup differences, all variables increased their effect size from pre-test to pos-test in favor of the experimental group, with high effect sizes in knowledge, accomplish, and experience motivation, amotivation, competence, relationship, personal and social responsibility, SDI and BPNs (>0.80); in the pre-test these values either had a medium effect (0.5–0.8) or a small effect (0.2–0.49). 

### 3.3. Differences in the Experimental Group According to the Educational Stage

Considering the experimental group and differentiating according to the educational stage (last two years of Elementary or Secondary school), we found the results that can be seen in Table 3 and Table 4. In this sense, Table 4 highlights that, in the pre-test, the differences were found in experience motivation, identified regulation, introjected regulation, competence, social relationships, and social responsibility. All these variables were significant at *p* < 0.05 (except for experience motivation at *p* < 0.01), with a value close to significance in the SDI (*p* = 0.056), and with higher results in Elementary than Secondary school. According to the d-Cohen effect, only autonomy, amotivation and external regulation had no differences, and experience motivation was the only variable whose effect size was considered medium (0.5).

In the post-test, statistically significant differences were observed for experience motivation, external regulation, amotivation, personal responsibility, and SDI. In this case, the external regulation and amotivation variables were higher in Secondary school. The values of d-Cohen effect were near to medium effect in experience_motivation, external regulation, personal responsibility, and SDI (0.4–0.5), similar to the *p*-value results, excluding amotivation.

Finally, Table 5 compares the experimental group with the results of the intervention. Regarding the Elementary school group, statistically significant differences were obtained that were not found in the Secondary school group in knowledge motivation, experience motivation, accomplish motivation, amotivation, social responsibility, and BPNs. In contrast, the Secondary school group only obtained *p* values < 0.01 for external regulation, which was not found in the elementary School group. According to d-Cohen test, the values for Secondary students were very small for all variables, but for Elementary students, a small effect was found in amotivation, autonomy, competence, personal and social responsibility, SDI, and BPNs.

## 4. Discussion

The main objective of this study was to verify the effects of the TPSR on different variables following the SDT, specifically, the levels of motivation, basic psychological needs, and responsibility of the students. It was intended to analyze the differences in the application of the model, taking into account whether the students were from Elementary school or Secondary school.

Considering the first proposed hypothesis, it is noteworthy that the experimental group, despite having obtained significantly higher values in almost all the variables in the pre-test, was the one that managed to improve these values at the end of the intervention. In contrast, the control group did not significantly modify any of the variables studied. In this sense, it is noteworthy that positive results were found in the motivation variable, obtaining higher values in the most self-determined regulation and a reduction in amotivation. This is in line with other authors who have applied this methodology and found improvements in intrinsic motivation [36,54], which also can be extended to the participating teachers, having greater satisfaction with their own teaching, and allowing them a greater transfer of autonomy to the students [7].

In turn, the BPNs improved, especially those of autonomy and competence perception, but this was not the case in the need for social relationships, which did not change. These results are consistent with different studies, such as those by Manzano-Sánchez et al. [18], where autonomy was the psychological need that benefited the most from the application of the TPSR, probably due to the fact that level 3 of the model is called “Autonomy” and TPSR strategies include the progressive surrender of autonomy as a basic pillar of the model. The perception of competence also improved, as in the study by Menéndez-Santurio et al. [41], where applying the TPSR in 143 Secondary school students obtained improvements in this need and also, unlike in our study, in those of relationship. This may be because, in the indicated works, the TPSR was applied only to PE, a subject where social relationships are practically necessary and where this methodology can help to promote said relationships due to the characteristics of the subject [64].

On the other hand, responsibility levels increased after the application of the model. The differences were only statistically significant in social responsibility, as in the study by Pérez-Ordás et al. [43], and contrary to Manzano-Sánchez et al. [18], where improvements were seen in personal responsibility. Other studies that have applied the TPSR have found improvements in both levels of responsibility [33,41], so it is interesting to indicate that there is still work to be done to reach conclusions on this variable. It is noteworthy that this methodology has not only shown very favorable results in psychomotor [46] and psychological aspects such as motivation and self-efficacy in exercise [47], but researchers such as Melero-Cañas et al. [65] analyzed the application of the TPSR in relation to the physical condition of Secondary school students and also improvements with a healthy lifestyle. The intervention implemented by Martins et al. [66] demonstrated that sport represents a unique opportunity within the educational process to establish values, beliefs, attitudes, and practical habits of relationships and cooperation that generate social responsibility in youngsters. Furthermore, Carreres-Ponsoda et al. [67] conclude that the TPSR model has the potential to be adapted and implemented also with flexibility in youth sport competition contexts in order to improve personal and social responsibility, prosocial behavior and self-efficacy. Finally, the study by García-Castejón et al. [4] is very interesting given that they found improvements both in the most self-determined motivation, as well as in the satisfaction of BPNs and in responsibility in a Secondary school by combining the TPSR with Teaching Games for Understanding in PE. This study should be indicated in context with other hybridizations performed with the TPSR, such as the use of the Sport Education Model, the most widespread model and one in which very positive results have been seen [41,68].

Based on the second of the hypotheses, the results obtained indicate higher values in the variables of motivation, satisfaction of BPNs, and responsibility for Elementary school students. Other authors have obtained similar results [33,69], where the Elementary education stage is shown to be the ideal one at which to work on motivational aspects.

Finally, this is the second study where the differences have been seen when applying the TPSR between two educational stages as a third hypothesis. In this sense, the research by Sánchez-Alcaraz [33] corroborated our results by applying the TPSR in PE, where the results of the intervention were better for Elementary school students. Although, unlike his study, in the present investigation the model was applied in PE and in other school subjects, and not only in PE. Other studies have verified the results in the sample taking into account students from both stages, but without differentiating between Elementary and Secondary school [7,36]. Therefore, there is still much to be investigated in this field of TPSR within the general educational context.

As the main limitations of this research, it should be noted that although both groups were quite similar in sample size, an attempt could have been made to obtain a larger sample and differentiate between the students who applied the model only in PE and in other subjects. In turn, it could have been considered to include the point of view of families or teachers when collecting the data to compare the results. On the other hand, the application of the TPSR was always of a teaching percentage greater than 60% of the total, but it would have been interesting to be able to apply it with all the participating teachers as a center project. It is also noteworthy that the pre-test values were higher in the experimental group and it could influence the results. Finally, the intervention was carried out for five months, and it would have been interesting to be able to carry it out during a total academic year.

Future studies could corroborate the results of the present study by applying the TPSR and even experimenting on the hybridization of methodologies within the general educational context. Considering different socio-educational levels can also be interesting in order to assess the variables under study. Additionally, it would be useful to understand the influence of the TPSR on variables such as emotional intelligence [70] or resilience as psychological variables, in addition to being able to extend the studies longitudinally, monitoring them over time.

## 5. Conclusions

The application of the TPSR in the educational context may be suitable both for teaching in Elementary school and in Secondary school students. The results obtained indicate that Elementary school students may have better results for self-determined motivation and responsibility after the intervention of TPSR compared with Secondary students. In turn, they are the ones who obtain the greatest benefits in the application of the TPSR, since the results of the intervention indicated improvements in both experimental groups, although these results were higher for the youngest students.

## Figures and Tables

**Table 1:** Descriptive analysis and correlations.

		*M*	*SD*	*Ran*	*S*	*k*	2	3	4	5	6	7	8	9	10	11	12	13	14
1	Knowledge_M	5.57	1.17	1-7	−0.949	0.61	0.760 **	0.759 **	0.584 **	0.605 **	0.343 **	−0.205 **	0.583 **	0.520 **	0.504 **	0.594 **	0.489 **	0.677 **	0.655 **
2	Accomplish_M	4.79	1.32	1-7	−0.469	−0.211	1	0.654 **	0.513 **	0.573 **	0.248 **	−0.239 **	0.633 **	0.461 **	0.484 **	0.567 **	0.462 **	0.680 **	0.647 **
3	Experience_M	5.55	1.24	1-7	−0.855	0.176		1	0.597 **	0.722 **	0.444 **	−0.244 **	0.471 **	0.509 **	0.454 **	0.566 **	0.455 **	0.652 **	0.581 **
4	Identified_R	5.66	1.07	1-7	−0.741	0.198			1	0.506 **	0.549 **	−0.334 **	0.406 **	0.425 **	0.384 **	0.475 **	0.414 **	0.682 **	0.493 **
5	Introjected_R	5.49	1.17	1-7	−0.788	0.126				1	0.457 **	−0.125 *	0.440 **	0.409 **	0.352 **	0.453 **	0.360 **	0.404 **	0.488 **
6	External_R	5.89	1.07	1-7	−1018	0.589					1	−0.146 **	0.224 **	0.228 **	0.209 **	0.225 **	0.207 **	0.250 **	0.268 **
7	Amotivation	1.76	1.17	1-5	1.739	2.408						1	−0.135 **	−0.242 **	−0.218 **	−0.384 **	−0.305 **	−0.801 **	−0.238 **
8	Autonomy	3.52	0.8	1-5	−0.237	−0.055							1	0.560 **	0.463 **	0.544 **	0.429 **	0.455 **	0.835 **
9	Competence	3.88	0.65	1-5	−0.29	−0.433								1	0.504 **	0.554 **	0.513 **	0.485 **	0.816 **
10	Relationship	4.18	0.78	1-5	−1.138	1.242									1	0.542 **	0.551 **	0.461 **	0.808 **
11	Personal_Res	5.21	0.73	1-6	−1.309	1.937										1	0.672 **	0.628 **	0.666 **
12	Social_Res	5.22	0.64	1-6	−0.897	0.401											1	0.513 **	0.605 **
13	SDI	7.06	3.9	//	−0.917	0.351												1	0.568 **
14	BPNs	3.86	0.61	1-5	−0.6	0.368													1

Legend: M = mean; SD = Standard deviation; Ran = Range; S = Skewness; *k* = Kurtosis; M = motivation; R = Regulation; Res = Responsibility; SDI = Self-determination index; BPNs = Basic Psychological Needs Index; ** = *p* < 0.01; * = *p* < 0.05.

**Table 2:** Main results of the intervention.

		Control				Experimental				Intergroup differences	
		Mean	SD	*p*-Value	d-Cohen	Mean	SD	*p*-Value	d-Cohen	*p*-Value	d-Cohen
**Knowledge_M**	**pre-test**	5.23	1.26	0.066	−0.05	5.87	0.99	0.003 **	0.18	0.001 **	0.57
	**post test**	5.17	1.28			6.05	0.97			0.001 **	0.90
**Accomplish_M**	**pre-test**	4.41	1.32	0.639	−0.02	5.13	1.23	0.013 **	0.19	0.001 **	0.56
	**post test**	4.39	1.3			5.34	1.15			0.001 **	0.80
**Experience_M**	**pre-test**	5.22	1.34	0.435	−0.02	5.85	1.06	0.001 **	0.24	0.001 **	0.53
	**post test**	5.19	1.38			6.09	0.97			0.001 **	0.89
**Identified_R**	**pre-test**	5.49	1.12	0.220	−0.04	5.81	1	0.001 **	0.24	0.003 **	0.30
	**post test**	5.45	1.15			6.05	0.97			0.001 **	0.61
**Introjected_R**	**pre-test**	5.3	1.23	0.889	0.04	5.66	1.08	0.001 **	0.07	0.004 **	0.31
	**post test**	5.35	1.28			5.74	1.09			0.002 **	0.36
**External_R**	**pre-test**	5.86	1.07	0.273	−0.01	5.91	1.08	0.381	0.01	0.701	0.05
	**post test**	5.85	1.03			5.92	1.08			0.330	0.06
**Amotivation**	**pre-test**	2.05	1.29	0.418	−0.05	1.5	0.99	0.007 **	−0.24	0.000 **	−0.48
	**post test**	1.99	1.25			1.3	0.6			0.000 **	−0.85
**Autonomy**	**pre-test**	3.44	0.79	0.178	0.01	3.59	0.8	0.001 **	0.31	0.019 *	−0.19
	**post test**	3.45	0.85			3.83	0.74			0.001 **	−0.49
**Competence**	**pre-test**	3.74	0.67	0.480	−0.07	4.01	0.6	0.001 **	0.47	0.001 **	0.43
	**post test**	3.69	0.77			4.28	0.55			0.001 **	1.03
**Relationship**	**pre-test**	3.96	0.86	0.131	−0.05	4.36	0.63	0.553	0.1	0.000 **	0.54
	**post test**	3.92	0.84			4.42	0.61			0.001 **	0.81
**Personal_Resp**	**pre-test**	5.00	0.81	0.063	−0.04	5.40	0.6	0.210	0.24	0.001 **	0.57
	**post test**	4.97	0.83			5.54	0.59			0.001 **	0.96
**Social_Resp**	**pre-test**	5.04	0.7	0.692	−0.01	5.39	0.53	0.001 **	0.20	0.001 **	0.57
	**post test**	5.03	0.71			5.5	0.57			0.001 **	0.85
**SDI**	**pre-test**	5.68	4.14	0.905	0.00	8.27	3.23	0.001 **	0.34	0.001 **	0.70
	**post test**	5.70	4.11			9.28	2.78			0.001 **	1.19
**BPNs**	**pre-test**	3.71	0.64	0.116	−0.03	3.99	0.55	0.002 *	0.33	0.001 **	0.47
	**post test**	3.69	0.7			4.17	0.51			0.001 **	0.91

Legend: M = Motivation; R = Regulation; Resp = Responsibility; SD = Standard deviation; SDI = Self-determination index; BPNs = Basic Psychological Needs Index; ** = *p* < 0.01; * = *p* < 0.05.

**Table 3:** Mean and Standard deviation pre-test.

	Elementary School		Secondary School			
	M	SD	M	SD	*p*-Value	d-Cohen
Knowledge_Motivation	5.95	0.87	5.61	1.28	0.201	−0.31
Accomplish_Motivation	5.20	1.16	4.91	1.41	0.233	−0.22
Experience_Motivation	5.98	0.97	5.42	1.23	0.004 **	−0.50
Identified_Regulation	5.88	1.01	5.6	0.92	0.022 *	−0.29
Introjected_Regulation	5.75	1.03	5.36	1.19	0.037 *	−0.35
External_Regulation	5.92	1.09	5.85	1.05	0.625	−0.07
Amotivation	1.49	0.96	1.52	1.09	0.812	0.03
Autonomy	3.60	0.79	3.55	0.86	0.735	−0.06
Competece	4.07	0.55	3.81	0.71	0.014 *	−0.41
Relationship	4.44	0.55	4.12	0.82	0.019 *	−0.45
Personal_Responsibility	5.47	0.49	5.18	0.83	0.098	−0.42
Social_Responsibility	5.44	0.5	5.23	0.61	0.031 *	−0.37
SDI	8.48	3.08	7.58	3.63	0.056	−0.27
BPNs	4.04	0.5	3.83	0.67	0.122	−0.35

Legend M = mean; SD = Standard deviation; SDI = Self-determination index; BPNs = Basic Psychological Needs Index; ** = *p* < 0.01; * = *p* < 0.05.

**Table 4:** Mean and Standard deviation post-test.

	Elementary School		Secondary School			
	M	SD	M	SD	*p*-Value	d-Cohen
Knowledge_Motivation	6.08	0.96	5.98	1.02	0.659	−0.10
Accomplish_Motivation	5.42	1.08	5.15	1.33	0.255	−0.22
Experience_Motivation	6.19	0.9	5.78	1.11	0.014*	−0.40
Identified_Regulation	6.06	0.97	6.00	0.98	0.613	−0.06
Introjected_Regulation	5.71	1.08	5.85	1.11	0.325	0.13
External_Regulation	5.82	1.15	6.26	0.72	0.021 *	0.47
Amotivation	1.25	0.54	1.45	0.74	0.025 *	0.31
Autonomy	3.80	0.71	3.92	0.85	0.192	0.15
Competence	4.28	0.51	4.26	0.67	0.721	−0.03
Relationship	4.44	0.6	4.33	0.73	0.458	−0.16
Personal_Responsibility	5.62	0.43	5.29	0.89	0.06 **	−0.46
Social_Responsibility	5.55	0.48	5.33	0.77	0.137	−0.34
SDI	9.58	2.55	8.31	3.28	0.015 *	−0.43
BPNs	4.17	0.47	4.17	0.62	0.626	0.00

M = mean; SD = Standard deviation; SDI = Self-determination index; BPNs = Basic Psychological Needs Index; ** = *p* < 0.01; * = *p* < 0.05.

**Table 5:** Results of the intervention according to educational stage.

		Elementary School				Secondary School			
		M	SD	*p*-Value	d-Cohen	M	SD	*p*-Value	d-Cohen
**Knowledge_M**	**pre-test**	5.92	0.91	0.008 **	0.06	5.27	1.27	0.121	0.06
	**post test**	5.98	1.05			5.35	1.26		
**Accomplish_M**	**pre-test**	5.20	1.20	0.016 *	0.13	4.46	1.32	0.462	0.04
	**post test**	5.35	1.13			4.51	1.33		
**Experience_M**	**pre-test**	5.92	1.02	0.001 **	0.20	5.24	1.32	0.075	0.05
	**post test**	6.11	1.00			5.30	1.34		
**Identified_R**	**pre-test**	5.87	1.00	0.012 *	0.15	5.48	1.09	0.008 **	0.07
	**post test**	6.02	0.97			5.56	1.16		
**Introjected_R**	**pre-test**	5.66	1.10	0.609	−0.01	5.35	1.21	0.017 *	0.10
	**post test**	5.65	1.15			5.47	1.23		
**External_R**	**pre-test**	5.85	1.15	0.511	−0.04	5.91	1.00	0.002 **	0.05
	**post test**	5.80	1.16			5.96	0.95		
**Amotivation**	**pre-test**	1.49	0.96	0.002 **	−0.31	1.98	1.29	0.914	−0.03
	**post test**	1.25	0.55			1.94	1.20		
**Autonomy**	**pre-test**	3.61	0.77	0.007 **	0.24	3.44	0.82	0.008 **	0.11
	**post test**	3.79	0.75			3.53	0.85		
**Competece**	**pre-test**	4.06	0.57	0.001 **	0.36	3.74	0.68	0.001 **	0.05
	**post test**	4.26	0.54			3.78	0.79		
**Relationship**	**pre-test**	4.43	0.56	0.851	−0.02	3.96	0.86	0.145	0.02
	**post test**	4.42	0.62			3.98	0.85		
**Personal_Resp**	**pre-test**	5.46	0.51	0.119	0.27	5.00	0.82	0.857	0.00
	**post test**	5.59	0.46			5.00	0.86		
**Social_Resp**	**pre-test**	5.44	0.50	0.001 **	0.20	5.04	0.69	0.087	0.01
	**post test**	5.54	0.49			5.05	0.74		
**SDI**	**pre-test**	8.49	3.05	0.001 **	0.33	5.87	4.13	0.001 **	0.05
	**post test**	9.43	2.58			6.07	4.16		
**BPNs**	**pre-test**	4.03	0.50	0.001 **	0.26	3.71	0.65	0.114	0.07
	**post test**	4.16	0.51			3.76	0.71		

Legend: M = mean; SD = Standard deviation; SDI = Self-determination index; BPNs = Basic Psychological Needs Index; ** = *p* < 0.01; * = *p* < 0.05.

## Data Availability

Data are available by contacting the corresponding author with a reasonable request.

## References

[1] Royal Decree 157/2022, of March 1, Which Establishes the Organization and Minimum Teaching of Primary Education. https://www.boe.es/eli/es/rd/2022/03/01/157/con.

[2] Royal Decree 217/2022, of March 29, Which Establishes the Organization and Minimum Teaching of Compulsory Secondary Education. https://www.boe.es/eli/es/rd/2022/03/29/217/con.

[3] Organic Law 3/2020, of December 29, Which Modifies Organic Law 2/2006, of May 3, on Education. https://www.boe.es/eli/es/lo/2020/12/29/3.

[4] Garcia-Castejon G., Camerino O., Castaner M., Manzano-Sanchez D., Jimenez-Parra J.F., Valero-Valenzuela A. (2021). Implementation of a hybrid educational program between the model of personal and social responsibility (TPSR) and the teaching games for understanding (TGfU) in physical education and its effects on health: An approach based on mixed methods. Children.

[5] Del Blanco M. (2017). Self-concept and school motivation: A bibliographic review. Infad J..

[6] Hortigüela D., Pérez A., Calderón A. (2016). Effect of the teaching model on the physical self-concept of students in physical education. Retos.

[7] Manzano-Sánchez D., Valero-Valenzuela A. (2019). Implementation of a model-based program to promote personal and social responsibility and its effects on motivation, prosocial behaviors, violence and classroom climate in primary and secondary education. Int. J. Environ. Res. Public Health.

[8] Manzano-Sánchez D. (2022). Physical education classes and responsibility: The importance of being responsible in motivational and psychosocial variables. Int. J. Environ. Res. Public Health.

[9] Jiménez-Parra J.F., Manzano-Sánchez D., Camerino O., Prat Q., Valero-Valenzuela A. (2022). Effects of a hybrid program of active breaks and responsibility on the behavior of primary students: A mixed methods study. Behav. Sci..

[10] Manzano-Sánchez D., Gómez-Marmol A., Jiménez-Parra J.F., Gil Bohorquez I., Valero-Valenzuela A. (2021). Motivational profiles and their relationship with responsibility, school social climate and resilience in high school students. PLoS ONE.

[11] Sánchez-Alcaraz B.J., Ocana-Salas B., Gómez-Marmol A., Valero-Valenzuela A. (2020). Relationship between school violence, sports personship and personal and social responsibility in students. Apunts.

[12] Gómez-Marmol A., Sánchez-Alcaraz B.J., Valero-Valenzuela A., de la Cruz-Sánchez E. (2018). Perceived violence, sociomoral attitudes and behaviors in school contexts. J. Hu. Sport. Exerc..

[13] Carrasco C., Alarcón R., Trianes M.V. (2015). Efficacy of a psychoeducational intervention based on the social climate, perceived violence and sociometrics in primary school students. Rev. Psychodidact..

[14] Pedreño N.B., Férriz-Morel R., Rivas S., Almagro B., Sáenz-López P., Cervelló E., Moreno-Murcia J.A. (2015). Sport commitment in adolescent soccer players. Motricidade.

[15] Chang Y.K., Chen S., Tu K.W., Chi L.K. (2016). Effect of autonomy support on self-determined motivation in elementary physical education. J. Sports Sci. Med..

[16] Escartí A., Gutiérrez M., Pascual C. (2011). Psychometric properties of the Spanish version of the Personal and Social Responsibility Questionnaire in physical education contexts. Rev. Psicol. Deporte.

[17] Lee O., Kim Y., Kim B. (2012). Relations of perception of responsibility to intrinsic motivation and physical activity among Korean middle school students. Percept. Mot. Skills.

[18] Manzano-Sánchez D., Valero-Valenzuela A., Conde-Sánchez A., Chen M.-Y. (2019). Applying the Personal and Social Responsibility Model-Based Program: Differences according to gender between basic psychological needs, motivation, life satisfaction and intention to be physically active. Int. J. Environ. Res. Public Health.

[19] Ryan R.M., Deci E.L. (2017). Self-Determination Theory: Basic Psychological Needs in Motivation, Development, and Wellness.

[20] Zheng W., Shen H., Belhaidas M.B., Zhao Y., Wang L., Yan J. (2023). The relationship between physical fitness and perceived well-being, motivation, and enjoyment in chinese adolescents during physical education: A preliminary cross-sectional study. Children.

[21] Deci E.L., Ryan R.M. (2013). Intrinsic Motivation and Self-Determination in Human Behavior.

[22] Deci E.L., Ryan R.M. (2012). Self-determination theory in health care and its relations to motivational interviewing: A few comments. Int. J. Behav. Nutr. Phys. Act..

[23] Ryan R.M., Deci E.L., Deci E.L., Ryan R.M. (2002). Overview of self-determination theory: An organismic-dialectical perspective. Handbook of Self-Determination Research.

[24] White R.L., Bennie A., Vasconcellos D., Cinelli R., Hilland T., Owen K.B., Lonsdale C. (2021). Self-determination theory in physical education: A systematic review of qualitative studies. Teach. Teach. Educ..

[25] Vallerand R.J. (1997). Toward a hierarchical model of intrinsic and extrinsic motivation. Adv. Exp. Soc. Psychol..

[26] Merino-Barrero J.A., Valero-Valenzuela A., Belando-Pedreño N. (2017). The model of personal and social responsibility. Study variables associated with its implementation. EmásF.

[27] Moreno-Murcia J.A., Huéscar E., Cervelló E. (2013). Prediction of adolescents doing physical activity after completing secondary education. Span. J. Psychol..

[28] Wang L., Chen R. (2022). Psychological needs satisfaction, self-determined motivation, and physical activity of students in physical education: Comparison across gender and school levels. Eur. J. Sport Sci..

[29] Santos F., Neves R., Parker M. (2020). Future pathways in implementing the teaching personal and social responsibility model in Spain and Portugal. Retos.

[30] Hellison D. (1985). Goals and Strategies for Teaching Physical Education.

[31] Jacobs J., Wright P. (2014). Social and emotional learning policies and physical education. Strategies.

[32] Escartí A., Pascual C., Gutiérrez M. (2005). Personal and Social Responsibility Through Physical Education and Sport.

[33] Sánchez-Alcaraz B.J., Gómez-Marmol A., Valero A., De la Cruz E. (2013). Implementation of a program to improve personal and social responsibility in physical education lessons. Mot. Eur. J. Hum. Mov..

[34] Bean C., Kendellen K., Forneris T. (2016). Moving beyond the gym exploring life skill transfer within a female physical activity based life skills program. J. Appl. Sport Psychol..

[35] Valero-Valenzuela A., López G., Moreno-Murcia J.A., Manzano-Sánchez D. (2019). From students’ personal and social responsibility to autonomy in physical education classes. Sustainability.

[36] Merino-Barrero J.A., Valero-Valenzuela A., Pedreño N.B., Fernandez-Río J. (2019). Impact of a sustained TPSR program on students’ responsibility, motivation, sportsmanship, and intention to be physically active. J. Teach. Phys. Educ..

[37] Escartí A., Llopis-Goig R., Wright P. (2017). Assessing the implementation fidelity of school-based teaching personal and social responsibility program in physical education and other subject areas. J. Teach. Phys. Educ..

[38] Fernández-Río J., Menéndez-Santurio J.I. (2017). Teachers and students’ perceptions of hybrid sport education and teaching for personal and social responsibility learning unit. J. Teach. Phys. Educ..

[39] Brown R., García-Arjona N. (2011). The Responsibility Model: Development of psychosocial aspects in socially disadvantaged youth through physical activity and sport. Rev. Psychol. Educ..

[40] Valero-Valenzuela A., Camerino O., Manzano-Sánchez D., Prat Q., Castañer M. (2020). Enhancing learner motivation and classroom social climate: A mixed methods approach. Int. J. Environ. Res. Public Health.

[41] Menéndez-Santurio J.I., Fernández-Río J. (2016). Violence, responsibility, friendship and basic psychological needs: Effects of a sports education and personal and social responsibility program. Rev. Psychodidact..

[42] Llopis-Goig R., Escartí A., Pascual C., Gutiérrez M., Marín D. (2011). Strengths, difficulties and aspects susceptible for improvement in the application of a program of personal and social responsibility in physical education. An evaluation based on the perceptions of its implementers. Cult. Educ..

[43] Pérez-Ordas R., Well P., Grao-Cruces A. (2020). Effects on aggression and social responsibility by teaching personal and social responsibility during physical education. J. Phys. Educ. Sport..

[44] Hsu W., Pan M., Shang I., Hsiao C. (2022). The Influence of Integrating Moral Disengagement Minimization Strategies into Teaching Personal and Social Responsibility on Student Positive and Misbehaviors in Physical Education. SAGE Open.

[45] Pavão I., Santos F., Wright P.M., Gonçalves F. (2019). Implementing the teaching personal and social responsibility model within preschool education: Strengths, challenges and strategies. Curric. Stud. Health Phys. Educ..

[46] Pan Y.H., Huang C.H., Lee I.S., Hsu W.T. (2019). Comparison of learning effects of merging TPSR respectively with sport education and traditional teaching model in high school physical education classes. Sustainability.

[47] Chunoh W.E.I., Ronghai S.U., Maochou H.S.U. (2020). Effects of TPSR integrated sport education model on football lesson students’ responsibility and exercise self-efficacy. Rev. De Cercet. Si Interv. Soc..

[48] Montero I., León O. (2002). Classification and description of research methodologies in Psychology. Int. J. Clin. Health Psychol..

[49] Tashakkori A., Teddlie C. (2010). Handbook of Mixed Methods in Social and Behavioral Research.

[50] World Medical Association (2013). World Medical Association Declaration of Helsinki: Ethical principles for medical research involving human subjects. JAMA.

[51] Vallerand R.J., Blais M.R., Brière N.M., Pelletier L.G. (1989). Construction and validation of the échelle de motivation en éducation (EME). Can. J. Behav. Sci..

[52] Núñez J.L., Martín-Albo J., Navarro J.G. (2005). Validity of the Spanish version of the Échelle de Motivation in Education. Psychothema.

[53] Curran P., West S., Finch F. (1996). The robustness of test statistics to nonnormality and specification error in confirmatory factor analysis. Psycho. Methods.

[54] Li W., Wright P., Rukavina P., Pickering M. (2008). Measuring students’ perceptions of personal and social responsibility and the relationship to intrinsic motivation in urban physical education. J. Phys. Educ. Recreat. Dance.

[55] Moreno-Murcia J.A., Gonzalez D., Chillon M., Parra N. (2008). Adaptation to physical education of the scale of basic psychological needs in the exercise. Rev. Mex. Psych..

[56] Moreno-Murcia J.A., Marzo J.C., Martínez-Galindo C., Marín L.C. (2012). Validation of psychological need satisfaction in exercise scale and the behavioral regulation in sport questionnaire to the Spanish context. RICYDE. Int. J. Sport Sci..

[57] Hellison D. (2011). Teaching Personal and Social Responsibility through Physical Activity.

[58] Manzano-Sánchez D., Conte-Marín L., Gómez-López M., Valero-Valenzuela A. (2020). Applying the personal and social responsibility model as a school-wide project in all participants: Teachers’ views. Front. Psychol..

[59] Escartí A., Gutiérrez M., Pascual C., Wright P. (2013). Observation of the strategies used by physical education teachers to teach personal and social responsibility. Rev. De Psicol. Del Deporte.

[60] Darling-Hammond L., Richardson N. (2009). Research review/teacher learning: What matters. Educ. Leadersh..

[61] Coulson C.L., Irwin C.C., Wright P.M. (2012). Applying Hellison’s responsibility model in a youth residential treatment facility: A practical inquiry project. Ágora.

[62] Hemphill M.A., Templin T.J., Wright P.M. (2015). Implementation and outcomes of a responsibility-based continuing professional development protocol in physical education. Sport Educ. Soc..

[63] Cohen J. (1988). Statistical Power Analysis for the Behavioural Sciences.

[64] Ventura T.C., Laborda J., Álvarez A.L. (2018). Physical education and social relations in primary education. Int. J. Educ. Psychol..

[65] Melero-Cañas D., Manzano-Sánchez D., Navarro-Ardoy D., Morales-Baños V., Valero-Valenzuela A. (2021). The Seneb’s Enigma: Impact of a hybrid personal and social responsibility and gamification model-based practice on motivation and healthy habits in physical education. Int. J. Environ. Res. Public Health.

[66] Martins P., González A.J., de Lima M.P., Faleiro J., Preto L. (2022). Positive development based on the Teaching of Personal and Social Responsibility: An intervention program with institutionalized youngsters. Front. Psychol..

[67] Carreres-Ponsoda F., Escartí A., Jiménez-Olmedo J.M., Cortell-Tormo J.M. (2021). Effects of a Teaching Personal and Social Responsibility Model intervention in competitive youth sport. Front. Psychol..

[68] González-Víllora S., Gospel E., Sierra-Díaz J., Fernández-Río J. (2018). Hybridizing pedagogical models: A systematic review. Eur. Phys. Educ. Rev..

[69] Manzano-Sánchez D. (2021). Differences between psychological aspects in primary education and secondary education. Motivation, basic psychological needs, responsibility, classroom climate, prosocial and antisocial behaviors and violence. Espiral.

[70] Rico-González M. (2023). Developing Emotional Intelligence through Physical Education: A Systematic Review. Percept. Mot. Ski..

